# *Lactobacillus acidipiscis* Induced Regulatory Gamma Delta T Cells and Attenuated Experimental Autoimmune Encephalomyelitis

**DOI:** 10.3389/fimmu.2021.623451

**Published:** 2021-02-19

**Authors:** Saisai Ren, Xiaorong Zhang, Hongbing Guan, Lihong Wu, Miao Yu, Dan Hou, Yongyong Yan, Xuechun Fang

**Affiliations:** ^1^Guangzhou Key Laboratory of Basic and Applied Research of Oral Regenerative Medicine, Department of Basic Science of Stomatology, Affiliated Stomatology Hospital of Guangzhou Medical University, Guangzhou, China; ^2^Guangzhou Medical University, Guangzhou, China

**Keywords:** *Lactobacillus acidipiscis*, γδ T cells, regulatory T cells, T helper cells, multiple sclerosis, experimental autoimmune encephalomyelitis

## Abstract

Multiple sclerosis is a chronic autoimmune disease involving the central nervous system, and shows a high disability rate. Its pathogenesis is complicated, and there is no good treatment. In recent years, with in-depth studies on the regulation of gastrointestinal flora, the relationship between the mammalian immune system and the intestinal flora has been extensively explored. Changes in the composition and structure of the gastrointestinal flora can affect the characteristics and development of the host immune system and even induce a series of central nervous system inflammation events. The occurrence and development of multiple sclerosis are closely related to the continuous destruction of the intestinal barrier caused by intestinal dysbacteriosis. In this study, we analyzed *Lactobacillus acidipiscis* in a mouse model of experimental autoimmune encephalomyelitis (EAE). We found that the amount of *L. acidipiscis* in the intestinal tract was inversely proportional to the progress of EAE development. In addition, the number of CD4^+^ FOXP3^+^ regulatory T cells in the mesenteric lymph nodes of mice increased significantly after the mice were fed with *L. acidipiscis*, and the differentiation of CD4^+^ T cells to Th1 and Th17 cells was inhibited. However, the protective effect of *L. acidipiscis* was lost in γδ T cell-deficient mice and hence was concluded to depend on the presence of regulatory γδ T cells in the intestinal epithelium. Moreover, including *L. acidipiscis* enhanced the development of Vγ1^+^γδ T cells but suppressed that of Vγ4^+^γδ T cells. In summary, our results demonstrated the ability of *L. acidipiscis* to induce generation of regulatory γδ T cells that suppress the development of the encephalomyelitic Th1 and Th17 cells and the progress of EAE.

## Introduction

More than 2.5 million people worldwide suffer from multiple sclerosis (MS), which is a chronic autoimmune disease affecting the central nervous system (CNS) and mainly occurring in young women with a high disability rate (1, 2). The pathogenesis of MS is very complex and its exact mechanism is unclear. Inflammation of the CNS and demyelination of the nerves are the main signs of MS. At present, MS is generally believed to be caused by autoreactive immune cells infiltrating the blood-brain barrier (BBB) with abnormal responses to autoantigens of the CNS, and myelin-specific CD4^+^ T cells are key to the occurrence of this disease (3). CD4^+^ helper T (Th) cells are considered to play the most important role in the pathogenesis of MS. Th1 and Th17 cells promote inflammation of the CNS, while Th2 and regulatory T (Treg) cells inhibit it. Experimental autoimmune encephalomyelitis (EAE) is a disease model mediated by specific sensitized CD4^+^ T cells, and is constructed by immunizing experimental animals with myelin sheath protein. This model is consistent with the induction method of MS, specifically combining myelin oligodendrocyte glycoprotein residues 35–55 (MOG_35−55_) with immunostimulant to induce the generation of pathogenic Th1 and Th17 cells (4).

Changes of intestinal microflora may lead to maladjustments of the immune response in the intestinal tract and other peripheral lymphoid sites including the CNS. There is increasing evidence that signal communication on the microbe-gut-brain response axis is closely related to the occurrence of MS, Parkinson's disease, Alzheimer's disease, depression, and other CNS diseases (5). Intestinal symbiotic bacteria that induce the generation of CD4^+^ T cells have been shown to change the severity of the demyelination of the CNS, and changing some bacterial groups in the intestinal tract could lead to a proinflammatory state, which in turn may lead to the development of autoimmune diseases, especially MS. Oral treatment of mice with antibiotics has been shown to reduce the severity of EAE by reducing inflammation and increasing accumulation of FOXP3 in mesenteric and cervical lymph nodes (6). Our results in 2016 demonstrated that the attenuation of EAE seen following CD44 gene deletion in mice, i.e., in CD44 knockout (CD44KO) mice, may result from alterations in the gut microbiota and short-chain fatty acids (SCFAs). Furthermore, our studies also demonstrated that phenotypes of gene knock-out animals in general may be shaped by gut microbiota (7).

*Lactobacillus acidipiscis* is an aerobic gram-positive lactobacillus acidophilus, and was recently discovered to be present in the lungs of healthy people, with this presence related to the function of pulmonary γδ T cells (8). In the intestinal epithelial lymphocytes (IELs) of mice, specifically in the duodenum and jejunum, the percentage of T cells consisting of γδ T cells was observed to be as high as 70%, much higher than that of αβ T cells (9). More interestingly, γδ T cells constitute the main target of intestinal bacteria. Aerobic bacteria or facultative anaerobes can activate γδ T cells more effectively than can anaerobes. One of the effectors produced by γδ T cells has been found to be IL-17, and hence these T cells are called IL-17-producing γδ T (γδ17 T) cells. In EAE, γδ17 T cells have shown both positive and negative effects, namely on the one hand aggravating the disease by increasing the quantity of Th 17 cells, but also on the other hand alleviating the disease by promoting apoptosis of Th17 cells and enhancing the function of Treg cells. The positive and negative effects of γδ17 T cells may be related to the heterogeneity of γδ T cells. The expression of CCR5 in Vγ1^+^ γδ17 T cells can enhance the function of Treg cells and induce the apoptosis of αβ T cells through FAS-FASL signal transduction. However, the expression of CCR6 in Vγ4^+^ γδ17 T cells promotes the generation of pathological Th17 cells (10, 11). Therefore, inducing protective regulatory γδ T cells has become a new strategy for treating MS. Studies have shown that intestinal bacteria can stimulate macrophages and dendritic cells to produce IL-1 and IL-23, and activate γδ17 T cells via the guanosine exchange factor 1 (VAV1) signaling pathway. According to the composition of T cell receptors, the peripheral γδ T cells in mice can be divided into two main subsets: Vγ1^+^ and Vγ4^+^ (12). However, Vγ1^+^ γδ T cells have been shown to produce more Th2-type cytokines such as IL-4, while Vγ4^+^ γδ T cells have been shown to preferentially produce IL-17A. In an experiment involving PMA/ionomycin-activated Vγ1^+^ and Vγ4^+^ γδ T cells, 20 differentially expressed genes were identified in the two cell subtypes, with most of these genes related to cytokines, cell differentiation, transcription and translation (13). In addition, intestinal probiotics were shown to produce protective short-chain fatty acids (SCFAs) such as acetate, propionate and butyrate acids (14), which have been shown to regulate the immune balance of intestinal and extraintestinal lymphoid tissues and organs, induce the differentiation of CD4^+^ T cells from Th1/Th17 cells to Th2/Treg cells, and induce the production of regulatory dendritic cells (DCs) and migration of Treg cells to gut-related lymphoid tissues (GALTs).

In rodent models, discrepancies in gut microbiota were found to be associated with in some cases susceptibility to EAE and other cases resistance to EAE (15). Previous studies from our laboratory showed that CD44 may also regulate inflammation, in as much as CD44 deficiency inhibits proinflammatory Th1 and Th17 cells while promoting CD4^+^ Th2 and Treg cell differentiation (16). In fact, CD44 deficiency led to decreased inflammation and amelioration of an experimental form of EAE. In those studies, CD44 gene deletion led to the alteration of gut microbiome and attenuation of EAE with simultaneously increase of Treg as well as decrease of Th17 (7).

In our current study, we found that *L. acidipiscis* became significantly more abundant in the intestinal flora of the EAE-resistant (CD44KO) mice, having become the predominant lactobacillus, and the number of γδ T cells in the intestinal tissue increased significantly. *L. acidipiscis* induced resistance of otherwise EAE-susceptible mice to EAE by inducing the proliferation of protective Treg cells and inhibiting the differentiation to Th1 and Th17 cells. In addition, *L. acidipiscis* was shown to be able to induce the generation of Vγ1^+^ γδ T cells having inhibitory effects on MS, with these T cells denoted as regulatory γδ T cells. In the absence of these regulatory γδ T cells, *L. acidipiscis* did not protect mice from EAE. Neither a clinical correlation between *L. acidipiscis* and MS nor its application in the clinical treatment of MS was investigated in the current work, but the experimental results that were obtained should provide the basis for further research in this field.

## Materials and Methods

### Mice and Reagents

Specific-pathogen-free (SPF) C57BL/6 female mice that were 6–8 weeks old were purchased from Guangdong Medical Laboratory Animal Center. Also, SPF CD44KO female mice that were 6–8 weeks old were purchased from Jackson Laboratory and TCR δ^−/−^ female mice that were 6–8 weeks old were purchased from Shanghai Model Organisms. All experimental animals were maintained under specific pathogen-free conditions at Guangzhou Medical University. All animal experiments were conducted under the protocols approved by and in accordance with the guidelines of the Institute Animal Care and Use Committee of the University. Myelin oligodendrocyte glycoprotein (MOG_35−55_) peptide was purchased from GL Biochem (Shanghai, China). Pertussis toxin was purchased from Tocris. Incomplete Freund's adjuvant was purchased from Sigma. Antibodies and isotypes were purchased from eBioscience and BioLegend. Cell stimulation cocktail (plus protein transport inhibitors, 500×) was purchased from eBioscience.

### Microbial Analysis in the Intestinal and *Lactobacillus acidipiscis* Culture

Fecal samples of EAE mice, specifically of CD44KO (KO) mice and C57BL/6 wild-type (WT) mice, were collected according to the procedure described previously (17). The microbial community of mouse intestinal contents was further analyzed using 16S rDNA. 16S rRNA amplicons were generated for the V3-V4 hypervariable regions of the fecal samples. Moreover, the *Lactobacillus acidipiscis* 10851 strain and *Escherichia coli* (*E. coli*) used in this study were obtained from the China Center of Industrial Culture Collection (CICC, Beijing, China). The strains were amplified in MRS liquid bacterial medium at 37°C for 24 h, and *L. acidipiscis* 10851 or *E. coli* solution was obtained. The solution was subjected to centrifugation at 3,000 g for 5 min, and the resulting supernatant was discarded to obtain the *L. acidipiscis* 10851 or *E. coli* precipitate. Each precipitate was suspended in sterile phosphate-buffered saline (PBS, pH 7.4), and subjected to another centrifugation at 3,000 g for 5 min. Then, the resulting supernatant was discarded, and the number of bacteria in the remaining liquid was counted after repeated washing. Final bacterial dilutions were carried out according to bacterial colony forming units (cfu/mL) at the indicated bacterial cell densities in different buffers or media.

### Collection of Feces and Analysis of SCFAs

The fecal contents were collected from C57BL/6 mice fed with *E. coli* or *L. acidipiscis* before being subjected to MOG_35−55_ peptide immunization. Feces were collected on day 15 of the immunization as described previously (18). Fecal contents (100 mg) were acidified with 25% metaphosphoric acid for 30 min on ice and then centrifuged at 12,000 g for 15 min at 4°C. Supernatants were filtered using Ultra free MC columns (0.22 μm GVDurapore, ThermoFisher Scientific) at 12,000 g for 4 min at 4°C. And then the eluates were analyzed using a GC-FID instrument.

### Probiotic Protection and EAE Induction

C57BL/6 mice (20 animals) were randomly divided into four groups: C57BL/6 mice that received fecal transfer from C57BL/6 mice, those from CD44 KO mice, and *E. coli* and *L. acidipiscis* protection groups (using 10851 strain). Moreover, TCR δ^−/−^ mice (10 animals) were randomly assigned to two groups: *E. coli* and *L. acidipiscis* protection groups.

Before carrying out the fecal transfers, recipient mice were treated with streptomycin and ampicillin to deplete endogenous gut microbiota. As described previously (19), all mice were given antibiotics (penicillin and streptomycin, 1 mg/mL aqueous solution, 100 μl) by carrying out oral gavage for two consecutive days. Twenty-four hours after the second antibiotic feeding, C57BL/6 mice were fed fecal solution (200 μl) collected from C57BL/6 or CD44KO mice for two consecutive days. Using a procedure similar to that used for the fecal transfer groups, some of the mice were given *E. coli* and others *L. acidipiscis* (6 × 10^8^ cfu/mL, 200 μl) using oral gavage for two consecutive days. At the same time, mice deficient in γδ T cells (TCR δ^−/−^) were fed *E. coli* or *L. acidipiscis* 10851 in the same manner.

EAE was observed to be induced in all mice by immunizing them with 150 μg of MOG_35−55_ in CFA-containing heat-killed *Mycobacterium tuberculosis* (strain H37Ra, 6 mg/mL) as described previously (20). Then, on days 0 and 2 post-immunization, the mice were treated intraperitoneally with, respectively, 200 and 400 ng of pertussis toxin. Mice were analyzed every day, and severity of EAE was scored using the 0–5 scoring grade: 0, asymptomatic; 1, tail tension loss; 2, unilateral hind limb paralysis; 3, paralysis of hind legs on both sides; 4, paralysis of forelimb; and 5, moribund.

### Detection of Th Subsets *in vivo* After *L. acidipiscis* Feeding

On the 15th day after the C57BL/6 mice were immunized with the MOG_35−55_ peptide, cells were isolated from the mesenteric lymph nodes of C57BL/6 mice after they were fed *E. coli* or *L. acidipiscis*. To investigated the frequency of Treg cells in the mesenteric lymph nodes, cells were stained with FITC-conjugated anti-mouse-CD4 (GK1. 5, eBioscience) and APC-conjugated anti-mouse FOXP3 antibody (FJK-16s, eBioscience). In addition, part of the cells isolated from the mesenteric lymph nodes were re-stimulated with MOG_35−55_ *in vitro* to observe the differentiation of Th1 and Th17. These encephalitogenic CD4^+^ T cells were cultured in RPMI1640 medium (Gibco BRL) with 10% FCS and MOG_35−55_ (30 μg/mL). Twenty-four hours later, the cells were collected and stained with FITC-conjugated anti-mouse-CD4 (GK1. 5, eBioscience), PE-conjugated anti-mouse IL-17A antibody (eBio17B7, eBioscience) and PerCP-conjugated anti-mouse IFN-γ antibody (XMG1. 2, eBioscience). The concentrations of IFN-γ, IL-17A, IL-10 and IL-13 in the supernatant were determined by using a commercial ELISA kit.

### γδT Activation and CD4^+^ T Differentiation *in vitro* With *L. acidipiscis* Stimulation

For γδT activation, flat-bottom 12-well plates were coated with 500 μl of purified anti-mouse TCR γ/δ antibody (UC7–13D5, 1 μg/ml, BioLegend) at 4°C overnight as described previously (21). Splenocytes were collected from EAE-C57BL/6 mice on day 15 post-immunization when EAE symptoms peaked, and B cells were removed from the population of splenocytes by carrying out magnetic separation using an EasySep FITC Selection Kit (Stemcell). The remaining cells were added to the Ab-coated wells and cultured in RPMI1640 medium (Gibco BRL) supplemented with 10% fetal calf serum and IL-2 (200 IU/ml) for 8 days. The resulting cells (2 × 10^6^ cells) were co-cultured with *E. coli* or *L. acidipiscis* (1 × 10^7^ cfu) at a ratio of 5:1 splenocyte for 3 days. Cells were collected and stained with FITC-conjugated anti-mouse-TCR Vγ1.1/ Cr4 antibody (2.11, Biolegend), and APC-conjugated anti-mouse TCR Vγ2 antibody (UC3-10A6, Biolegend).

CD4^+^ T differentiation *in vitro* was performed as previously described with minor modification (16). Briefly, splenocytes were prepared from EAE-C57BL/6 mice on day 15 post-immunization, co-cultured with *E. coli* or *L. acidipiscis* (1 × 10^7^ cfu) at a ratio of 5:1 splenocyte and 30 μg/ml of MOG_35−55_ for 24 h, followed by stimulation with Cell Stimulation Cocktail (plus protein transport inhibitors, 500×, eBioscience) for 5 h. Production of IL-4 and IL-17A in the CD4^+^ T cells were then detected by intracellular staining and flow cytometry.

### Intracellular Staining, Flow Cytometry and Cytokine Assays

For staining of intracellular IFN-γ, FOXP3, IL-4 and IL-17A, cells were stimulated for 5–6 h with Cell Stimulation Cocktail (plus protein transport inhibitors, 500 ×, eBioscience), which is a cocktail of phorbol 12-myristate 13-acetate (PMA) and ionomycin. The cells were harvested, washed twice with PBS, and analyzed for the presence of Treg and Th17 cells. The cells were then fixed, permeabilized, and stained with IFN-γ-PerCP, FOXP3-APC, IL-4-PE-Cyanine7 (11B11, eBioscience) and IL-17A-PE antibodies. Fluorescence signals were detected using a BD FACS Canto II flow cytometer (BD Biosciences, San Jose, CA, USA). Data were analyzed by using FlowJo (Tree Star, Ashland, OR, USA) software.

### Determination of Concentrations of Cytokines Using ELISA

The concentrations of IFN-γ, IL-17A, IL-10 and IL-13 in supernatants were determined by using a commercial ELISA kit (BioLegend) according to the manufacturer's instructions.

### Histopathology

Intestinal tissues were removed from mice as a result of subjecting heart to heparin-PBS perfusion and fixed in 10% paraformaldehyde. These intestinal tissues were then treated with GL3 antibody stain, and examined under an optical microscope in order to visualize γδ T cells.

### Statistical Analysis

Statistical difference between different groups was analyzed by performing the Student's *t*-test using Graph Pad Prism 6.2 software (GraphPad Software Inc, San Diego, CA, USA). The nonparametric data (EAE scoring) were analyzed using the Mann-Whitney U test. Values of *P* < 0.05 were considered to indicate statistical significance. The statistical analysis data are presented in the manuscript as mean ± SD or mean ± SEM.

## Results

### The Number of *L. acidipiscis* Bacteria Was Significantly Increased in EAE-Resistant Mice

**Figure 1:**
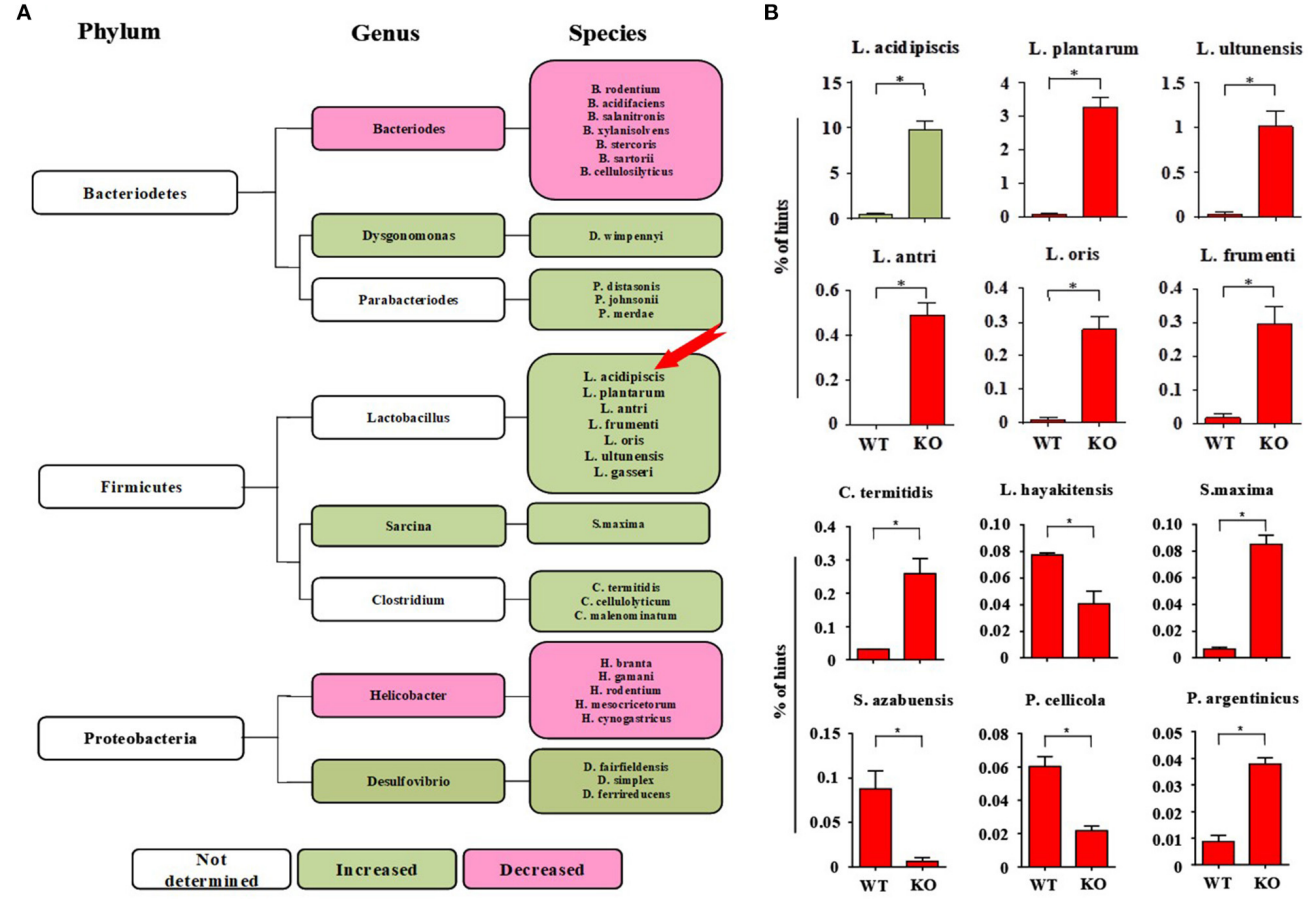
The number of *L. acidipiscis* bacteria was significantly increased in EAE- resistant mice. **(A)** Plots of the intestinal flora compositions of CD44KO and WT mice. The number of lactobacilli and number of desulphurized vibrio bacteria were higher than of WT mice in the intestinal tract of CD44KO mice, while those of bacteroidetes and helicobacter were lower. **(B)** The number of *L. acidipiscis* bacteria was significantly increased in CD44KO mice, and *L. acidipiscis* was the predominant lactobacillus in the intestinal flora of EAE- resistant mice. C57BL/6 wild-type and CD44KO mice are labeled as WT and KO, respectively. Data are shown as mean ± SD and are from a single experiment representative with three mice per experimental group, comparisons were made between EAE WTs (*n* = 3) vs. EAE KOs (*n* = 3). ^*^P < 0.05, Student's *t*-test.

On the 15th day after mice were immunized with MOG_35−55_ peptide, 16s rRNA V4 sequencing analysis of the intestinal flora of the mice was performed. We found that the intestinal flora composition of CD44KO mice was significantly different from that of WT. The number of *L. acidipiscis* in the intestines of the CD44KO mice was much higher than that in C57BL/6 mice (Figure 1A) (*p* < 0.05). The part of the intestinal flora compositions of the EAE-resisitant and WT mice are shown in Figure 1B. Significantly different compositions of intestinal bacteria strains in the CD44KO and WT group were observed. Also, significantly more *L. acidipiscis* bacteria than other lactobacilli were found in the CD44KO group, and *L. acidipiscis* was the predominant lactobacillus in the intestinal flora of the CD44KO mice.

### *L. acidipiscis* Induced the Production of Intestinal SCFA

**Figure 2:**
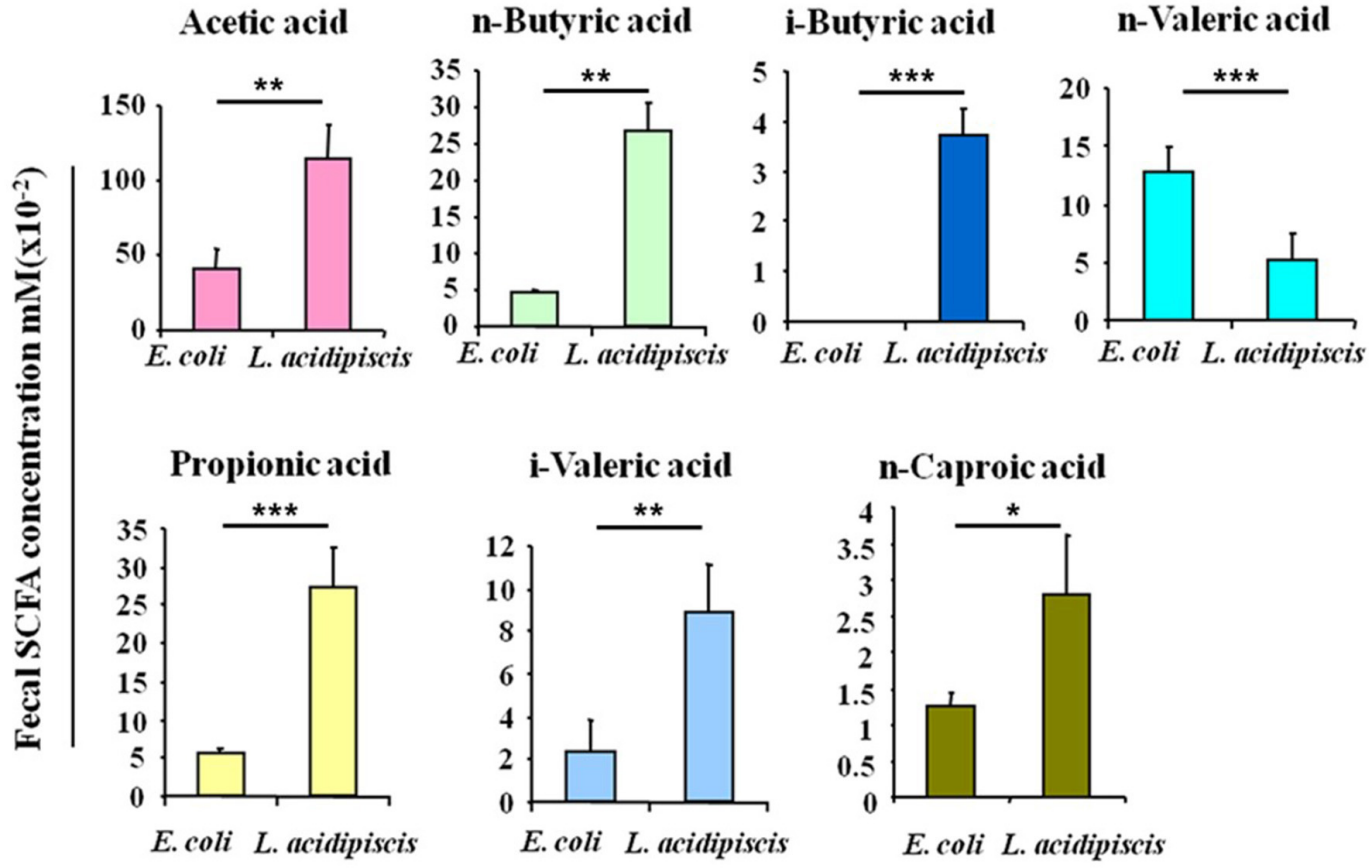
*L. acidipiscis* induced the production of intestinal SCFA. Concentrations of SCFAs in the fecal contents of EAE-WT mice that had been fed *E. coli* or *L. acidipiscis*. Data shown are mean ± SD (^*^P < 0.05, ^**^P < 0.01, ^***^P < 0.001, Student's *t*-test) and are from a single experiment representative with five mice per experimental group.

To explore the effect of *L. acidipiscis* on the content of SCFAs in the intestinal tracts of C57BL/6 mice that had been fed *L. acidipiscis*, an EAE model was established by immunizing some of the C57BL/6 mice with the MOG_35−55_ peptide, and treating other mice with *E. coli* as the control group. Mouse feces were collected on day 15 after the immunization with the MOG_35−55_ peptide, and the SCFA content in mouse feces was determined using the GC-FID method. The abundances and concentrations of acetic acid, n-butyric acid, i-butyric acid, n-valeric acid, propionic acid, i-valeric acid and n-caproic acid in each sample are shown in Figure 2. The amounts of acetic acid (*p* < 0.01), n-butyric acid (*p* < 0.01), i-butyric acid (*p* < 0.001), propionic acid (*p* < 0.001), i-valeric acid (*p* < 0.01) and n-caproic acid (*p* < 0.05) in the feces of C57BL/6 mice fed with *L. acidipiscis* were significantly higher than for those fed with *E. coli*. Furthermore, the data showed a significant difference (*p* < 0.001) in the concentration of n-valeric acid between the EAE-WT mice that received *E. coli* and those receiving *L. acidipiscis*, indicating that *L. acidipiscis* could induce protective immunophenotypes by synthesizing acetic acid and other SFCAs in the intestinal tracts of EAE-susceptible mice.

### *L. acidipiscis* Induced Resistance to EAE in Susceptible Mice

**Figure 3:**
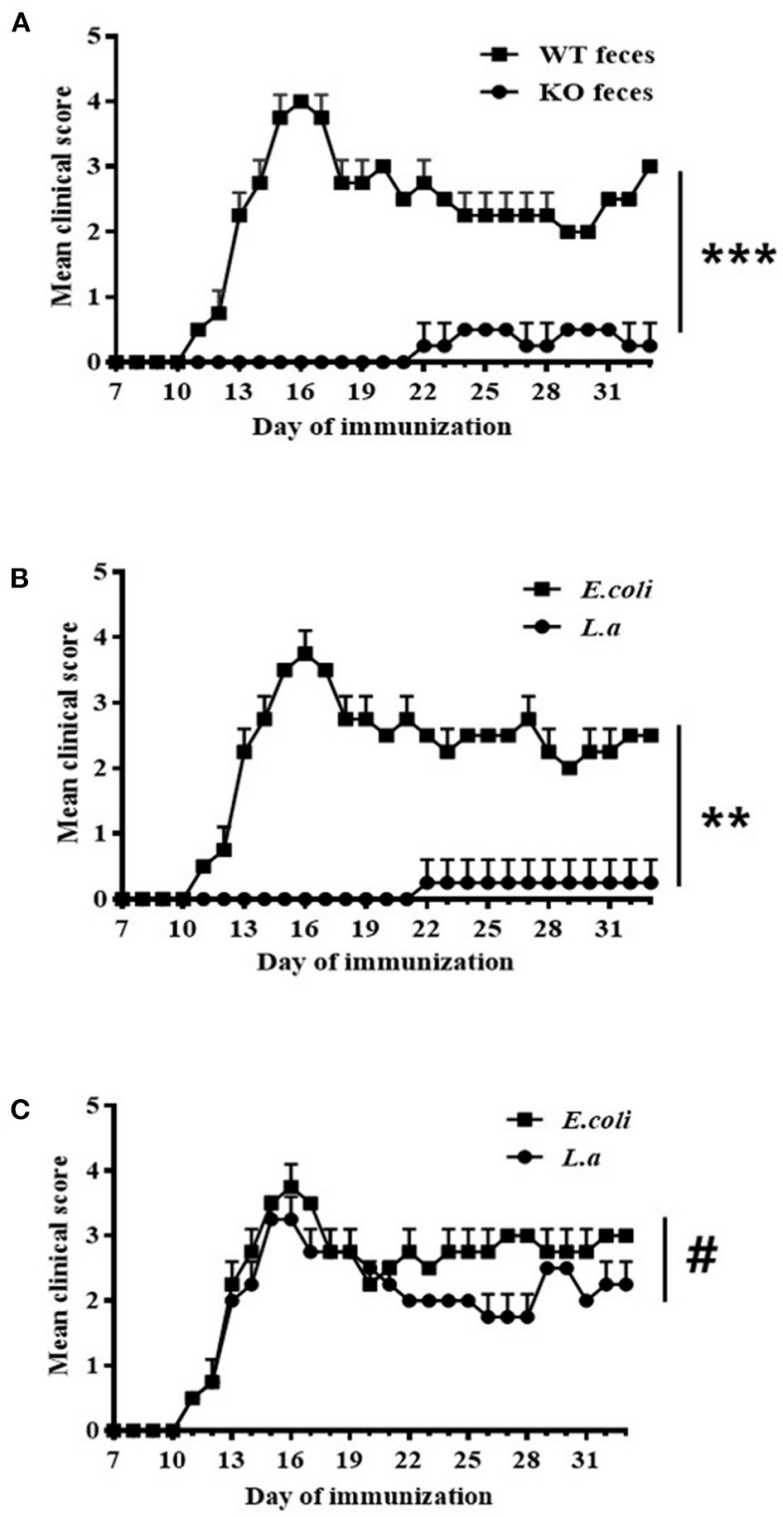
*L. acidipiscis* induced resistance to EAE in susceptible mice. **(A)** Results showing feces of CD44KO mice having induced resistance to EAE in WT receptor mice (WT feces: WT mice with EAE and that received feces from WT mice, *n* = 5; KO feces: WT mice with EAE and that received feces from CD44KO mice, *n* = 5). **(B)** Results showing *L. acidipiscis* having induced resistance to EAE in WT mice (*E. coli*: WT mice with EAE and that were fed *E. coli, n* = 5; *L. a*: WT mice with EAE and that were fed *L. acidipiscis, n* = 5). **(C)** *L. acidipiscis* did not produce resistance to EAE in TCRδ^−/−^ mice (*E. coli*: TCRδ^−/−^ mice with EAE and that were fed *E. coli, n* = 5; *L. a*: TCRδ^−/−^ mice with EAE and that were fed *L. acidipiscis, n* = 5). This result suggested the presence of γδT cells to be required for realizing the protective effect of *L. acidipiscis*. Clinical scores were recorded daily after EAE induction and fecal transfer. The values are shown as mean ± SEM. Data were analyzed using the Mann–Whitney *U* test. ^**^P < 0.01, ^***^P < 0.001, #*P* > 0.05.

Seven days before being immunized with the MOG_35−55_ peptide, WT (Figures 3A,B) and TCRδ^−/−^ (Figure 3C) mice were inoculated with various materials: some mice were inoculated with fecal material of CD44KO mice (Figure 3A), others with *L. acidipiscis* (Figures 3B,C), and still others with the fecal material of WT mic**e** (Figure 3A) or *E. coli* bacteria (Figures 3B,C), respectively, as controls. Feeding CD44KO mouse feces or an *L. acidipiscis* bacteria suspension to WT mice significantly inhibited the occurrence of EAE and reduced the degree of disease (Figures 3A,B). However, the occurrence of EAE was not significantly inhibited when *L. acidipiscis* was fed to mice with a deficiency of γδT cells (TCRδ^−/−^) (Figure 3C), suggesting that the protective effect of *L. acidipiscis* on susceptible mice requires the presence of γδT cells.

### *L. acidipiscis* Induced Treg Cell Development and Inhibited Pathological Th1 Cell and Th17 Cell Differentiation

**Figure 4:**
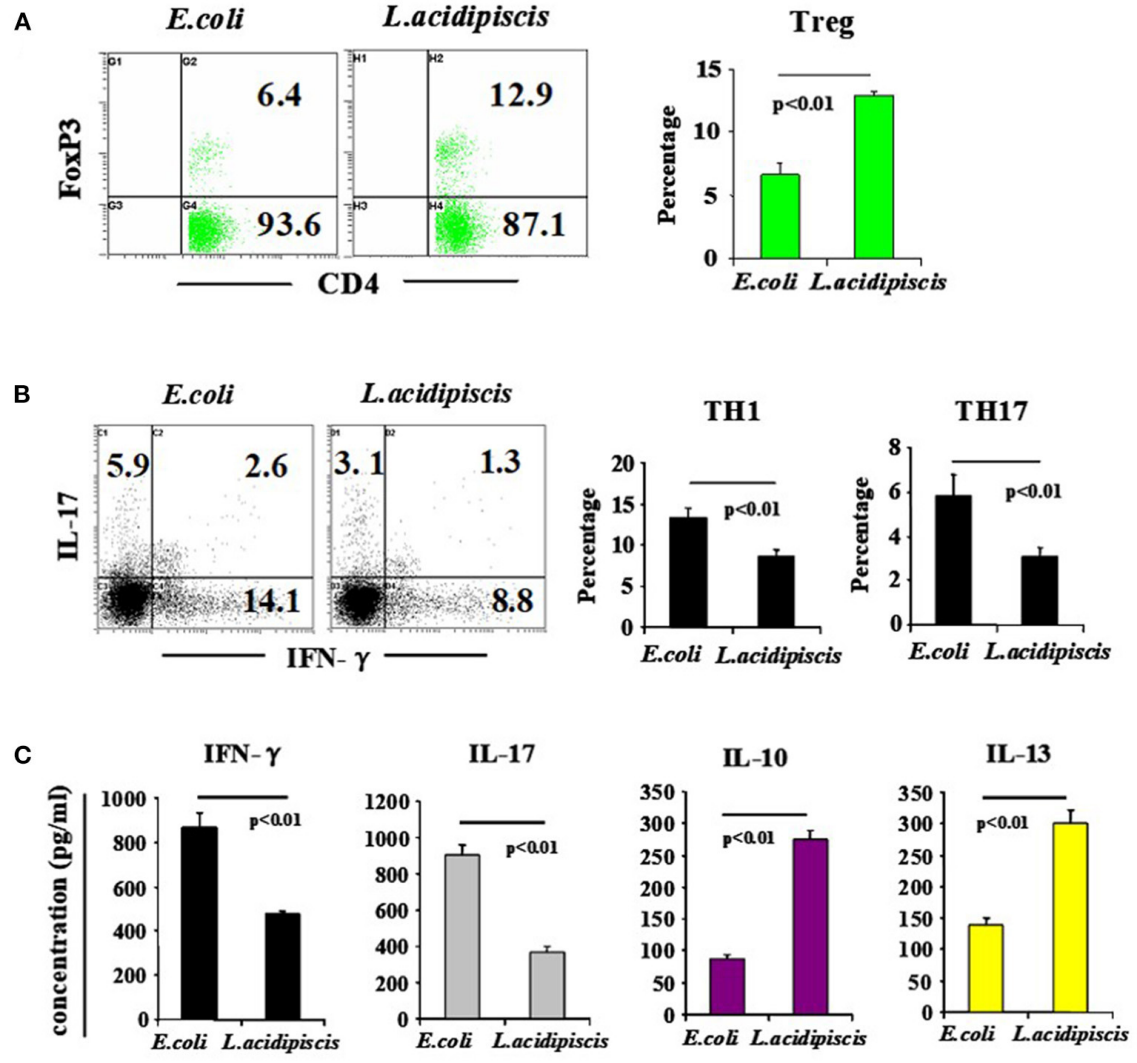
*L. acidipiscis* induced the development of Treg cells and inhibited pathological differentiation into Th1 and Th17 cells. Percentages of CD4^+^ T cells consisting of Treg, Th1 and Th17 cells in mesenteric lymph nodes of C57BL/6 mice fed with bacteria were determined on day 15 when EAE symptoms peaked. **(A)** FACS detection of CD4^+^ FOXP3^+^ Treg cells, and percentage of CD4^+^ T cells consisting of Treg cells. **(B)** FACS detection of Th1 and Th17 cells, and percentage in CD4^+^ T cells. **(C)** Cytokine concentration in cell culture supernatant. Representative flow cytometry plots were derived from a single experiment with 5 mice per experimental group and average percentage of each subset was expressed as mean ± SEM from three independent experiments. Statistical differences were determined by using Student's *t*-test, and data with *P* < 0.01 represent significant differences between the two groups.

On the 15th day after mice were immunized with MOG_35−55_ peptide, the number of Treg cells from mesenteric lymph nodes of C57BL/6 mice that were fed *L. acidipiscis* was significantly greater than that for C57BL/6 mice that were instead fed control material, and the differentiation of CD4^+^ T cells to Th1 and Th17 cells was inhibited. The production of protective IL-10 and that of IL-13 were each increased, while the production of pathological IFN-γ and that of IL-17A were each significantly decreased, indicating that *L. acidipiscis* may regulate the differentiation of cerebrospinal inflammatory CD4^+^ T cells and induce a deviation of the protective T cell immune response (Figure 4).

### The Number of γδ T Cells Increased Significantly in the Intestines of EAE- Resistance Mice

**Figure 5:**
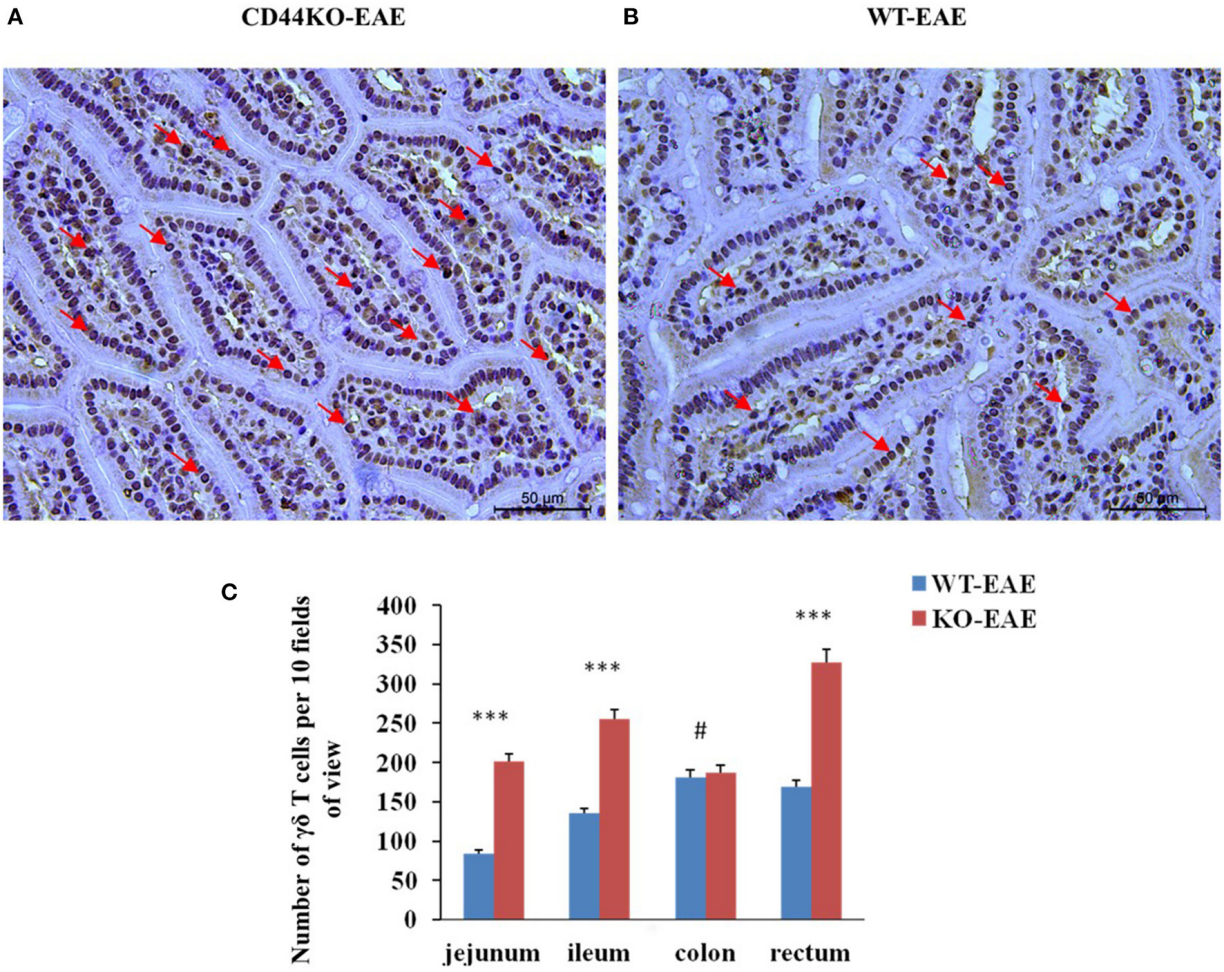
Significantly more γδ T cells were observed in the intestines of EAE-resistant mice. Representative histological appearances of jejunal tissues of CD44-EAE and WT-EAE mice on day 15 post-immunization, parts of γδ T cells with GL3 positive staining were pointed with red arrows. **(A)** Jejunum of a CD44KO-EAE mice. **(B)** Jejunum of a WT-EAE mice. **(C)** Mean numbers of γδ T cells in the jejunum, ileum, colon and rectum of 10 high-power field. Data from three separate experiments with five mice/group are presented as mean ± SEM. Data were analyzed with Student's *t*-test: ^***^P < 0.001, #*P* > 0.05.

We also set out to compare the numbers of γδ T cells in EAE-resistant mice and EAE-susceptible mice. For this purpose on day 15 post-immunization of mice with the MOG_35−55_ peptide, immunohistograms were acquired to detect the presence of γδ T cells (GL3 antibody staining positive) in the intestinal tissues of EAE-resistant mice (CD44KO-EAE) and EAE-susceptible mice (WT-EAE) (arrow in Figure 5). Significantly more γδ T cells were found in the intestinal tracts of CD44KO-EAE mice than in those of WT-EAE mice. We confirmed the resistance of CD44KO mice to EAE to be closely related to the presence of γδ T cells in the small intestinal epithelium of mice.

### *L. acidipiscis* Promoted the Development of Vγ1^+^ γδ T Cells and Inhibited Vγ4^+^ γδ T Cells *in vitro*

**Figure 6:**
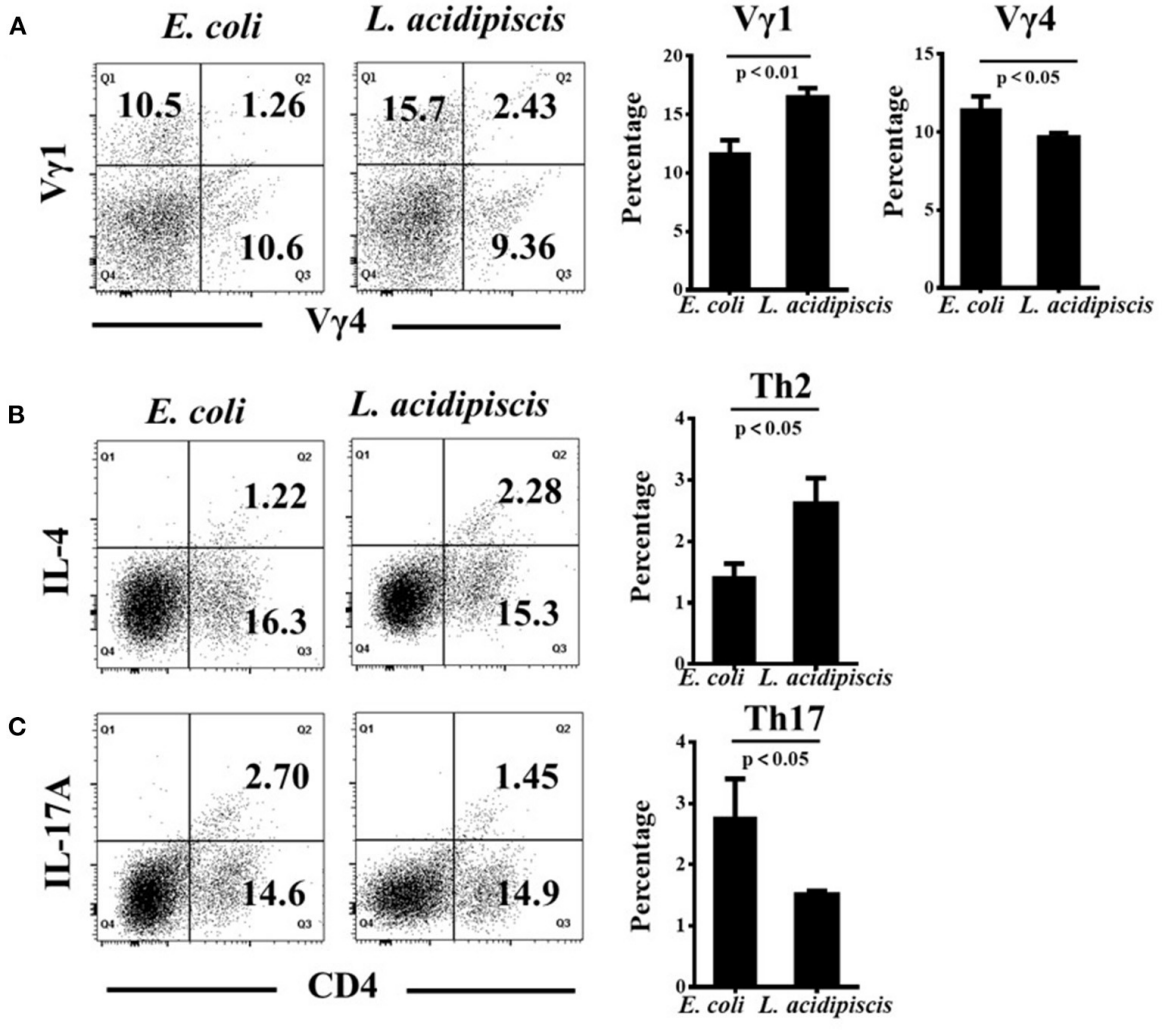
*L. acidipiscis* promoted the development of Vγ1^+^ γδ T cells and inhibited Vγ4^+^ γδ T cells *in vitro*. **(A)** The development of Vγ1^+^ γδ T cells was significantly enhanced while the development of Vγ4^+^ γδ T cells was significantly suppressed after they were co-cultured with *L. acidipiscis*. **(B)** The development of Th2 cells was significantly enhanced after they were co-cultured with *L. acidipiscis*. **(C)** The differentiation of Th17 cells was significantly inhibited after co-cultured with *L. acidipiscis*. Representative flow cytometry plots were derived from a single experiment with three mice per experimental group and average percentage of each subset was expressed as mean ± SEM from three independent experiments. Data were analyzed with Student's *t*-test, and data with *P* < 0.01, *P* < 0.05 represent significant differences between the two groups.

To investigate the differential induction of Vγ1^+^/ Vγ4^+^ γδ T cells and CD4^+^ Th cells by *L. acidipiscis*, we examined the differentiation of γδ T cells and encephalitogenic CD4^+^ T cells from the splenocytes of EAE mice. γδ T and CD4^+^ T cells were co-cultured with or without *L. acidipiscis* strains for 3 days (Figure 6). The *L. acidipiscis* strain induced a significant increase in the proportion of Vγ1^+^ γδ T cells, and it inhibited the proportion of Vγ4^+^ γδ T cells. In addition, the differentiation of Th2 cells was significantly enhanced after they were co-cultured with *L. acidipiscis* (Figure 6B), whereas the development of Th17 cells was significantly inhibited after they were co-cultured with *L. acidipiscis* (Figure 6C).

## Discussion

The cause of multiple sclerosis (MS) is unclear, and there are no effective methods for preventing and treating this disease. Immunology, genetics and histopathology data of patients with MS support the concept that autoimmunity plays a major role in the pathogenesis of the disease. Our current understanding of the pathogenesis of MS and that of its disease-causing mechanisms have mainly derived from the results of investigations using a classical mouse model; here, EAE is induced by performing subcutaneous immunization with an emulsion composed of a myelin component, such as MOG peptide, and complete Freund's adjuvant together with an administration of pertussis toxin. The activation of an autoimmune reaction and the production of myelin-specific CD4^+^ T cells have been identified as being key to the development of MS. Therefore, inducing protective immune phenotype constitutes an important strategy for treatment of MS.

Highly heterogeneous microbial populations reside in the gastrointestinal tracts of mammals, and are essential for the immune systems of the hosts to completely develop. Intestinal microbes determine the development of the host microbial population and immune system, which are in a complex balance; the genetic material of such microbes has recently been coined as the “microbiome.” Models for spontaneous EAE were found to be particularly useful for research on the role of gut microbiota in the induction of brain inflammation (22). The results of such research have indicated the signal communication of the gut bacteria-gut-brain response axis to be closely related to the occurrence of MS: when such animals are kept under germ-free conditions, no disease develops; but when their intestines are occupied by normal intestinal flora, disease is triggered. This model can be used to identify the bacterial components of intestinal flora, which can trigger and expand the pro-inflammatory T cells (Th1 and Th17 cells). Intestinal microorganisms come into contact with antigen-presenting cells (APCs) in the intestinal lumen or Peyer's patches. The APCs further present antigen to the naive T cells in the lamina propria. Naive T cells are further activated after migrating to mesenteric lymph nodes. The activated T cells diffuse through the blood and distal lymph nodes and migrate to the ileum propria (23). These results can help explain the mechanisms of chronic brain inflammation induced by antigen diffusion. Generally speaking, intestinal probiotics play an important role in MS, and can prevent and alleviate MS by inhibiting and treating inflammatory cells associated with this disease.

Gut-associated lymphoid tissue (GALT) is an important component of the immune system. Treg and Th17 cells induced by the intestinal tract are the characteristic T cells of the intestinal immune network (24). While IL-10 is responsible for maintaining the expression and function of FOXP3 in Treg cells (25). *B. adolescentis* IF1-03 has been shown to stimulate maturation of macrophages, producing higher levels of IL-10 and lower levels of IL-6 and TGF-β, features consistent with the upregulation of Treg cells in DSS-colitis mice *in vivo* and splenocytes *in vitro*. On the other hand, *B. adolescentis* IF1-11 has been shown to stimulate macrophages to secrete higher levels of IL-6 and TGF-β, and lower levels of IL-10, which promoted the differentiation of CD4^+^ T cells into Th17 cells (26). Therefore, as far as health and disease are concerned, specific bacterial species have been proven to have a significant impact on the differentiation of immune cell subsets and innate immune maturation (27). Specifically, probiotics and prebiotics have been reported to have positive immuno-equilibrium restorative effects. Probiotics contribute to the balance of cytokines, and can positively influence the progress of allergic and inflammatory diseases. Our results showed that compared with *E. coli, L. acidipiscis* increased the production of CD4^+^ FOXP3^+^ Treg cells, IL-10 and IL-13, and inhibited the production of Th1, Th17, IFN-γ and IL-17A. Clinical observations have shown beneficial clinical effects of probiotics, and probiotics strains may be used to treat inflammatory disease. Evidence has been presented to show that probiotics such as bifidobacterium and lactobacillus in the host can be involved in immune regulation by skewing of naive T cells toward Treg cells (28).

γδ T cells appear earlier than do αβ T cells in the development of thymus, mainly in the early stages of fetal development (29). Compared with αβ T cells, γδ T cells only represent a small number of T cell subsets (1–10%) in the peripheral blood, and are mainly present in epithelial tissue in the form of intraepithelial lymphocytes (IELs). γδ T cells have their own distinct characteristics, such as relatively low TCR diversity and being able to directly recognize antigen without the requirement of a presentation of the antigen that are distinct from those of αβ T cells (30). In addition, Benakis et al. demonstrated a reduction in ischemic brain damage in mice as a result of antibiotic-induced changes in the intestinal flora, and that the effects could be transmitted through fecal transplantation. Furthermore, intestinal dysbiosis has been found to alter immune homeostasis in the small intestine, leading to an increase in the number of Treg cells and a reduction in IL-17-positive γδ T cells through altered dendritic cell (DC) activity (31). These studies have focused on the role of the intestinal flora and gut-brain axis and IL-17 (+) γδ T cells in MS and EAE as both pathogenic and protective, their role in the CNS, the types of subsets and a possible role in Th17 inflammation.

The heterogeneity of phenotype and function of γδ T cells is not clearly demonstrated so far. Studies have shown activated Vγ1^+^ γδ T cells expressing relatively high levels of IL-4 and IL-5 (32), and Vγ4^+^ γδ T cells secreting relatively high amounts of IL-17A, IL-17F and IFN-γ (33). Vγ1^+^ γδ17 T cells and Vγ4^+^ γδ17 T cells are common subtypes of γδ17 T cells. Both cell types maintain the phenotype of producing IFN-γ, TNF-α, TGF-β and IL-10. While Vγ1^+^ γδ T cells produce more Th2-type cytokines such as IL-4 and IL-5, Vγ4^+^ γδ T cells preferentially produce IL-17 (34). These positive and negative effects may be related to the heterogeneity of γδ T cells. Therefore, the induction of protective regulatory γδ T cells by intestinal probiotics constitutes a new strategy for treating MS. Overall, our results provided evidence that *L. acidipiscis* could influnce the differention of γδ T cells and CD4^+^ T cells into diffrent subsets *in vitro*, altough the data was limited and lack of *in vivo* evidence. The further study was needed in clarification of the related questions.

## Conclusions

In this study, we found a negative correlation between *L. acidipiscis* in the intestinal tract and the progress of EAE. The resistance of CD44KO mice to EAE was related to the stimulation and activation of γδ T cells in small intestine epithelial tissues by *L. acidipiscis*. Here, we showed the presence of significantly more γδ T cells in the intestinal tracts of CD44KO-EAE mice than in those of WT-EAE mice. The resistance of CD44KO mice to EAE could be transmitted by intragastric administration of *L. acidipiscis* or feces transplanted from CD44KO mice. We analyzed the composition of intestinal flora and the levels of *L. acidipiscis* in EAE. Our results showed a negative correlation between the amount of intestinal *L. acidipiscis* and the progression of EAE, and showed the regulation of encephalitogenic CD4^+^ T cell differentiation by *L. acidipiscis* to be related to γδ T cells. Meanwhile in our experiments, *L. acidipiscis* suppressed *in vitro* the proliferation of Th1 and Th17 cells as well as the secretion of IFN-γ and IL-17A, and clearly promoted the development of Treg and Th2 cells. In contrast, EAE was not significantly inhibited when *L. acidipiscis* was fed to TCRδ^−/−^ mice. In summary, we have provided evidence for *L. acidipiscis* being a critical factor in the development of mouse Treg cells. In our experiments, *L. acidipiscis* was used to induce regulatory T cells to differentiate into protective cell subsets, thus EAE-susceptible mice obtaining the resistance to EAE. In addition, our results demonstrated an association between progression of MS and a decreased proportion of *L. acidipiscis* in the host intestinal tract, and demonstrated the ability of *L. acidipiscis* to target Vγ1^+^ and Vγ4^+^ γδ T cells and to interfere with the expression of IL-10, IL-13, IFN-γ and IL-17A.

## Data Availability Statement

The raw data supporting the conclusions of this article will be made available by the authors, without undue reservation.

## Ethics Statement

The animal study was reviewed and approved by Institutional Animal Care and Use Committee of Guangzhou Medical University.

## Author Contributions

HG contributed to conception and design of the study. SR and XZ performed the experiments. LW and DH carried out data analysis. All authors participated in drafting of the manuscript and critical revision of the draft and contributed to the article and approved the submitted version.

## Conflict of Interest

The authors declare that the research was conducted in the absence of any commercial or financial relationships that could be construed as a potential conflict of interest.
